# Supplementary material for: Glomerular Endothelial Cell-Derived miR-200c Impairs Glomerular Homeostasis by Targeting Podocyte VEGF-A
Source: Int J Mol Sci. 2022 Dec 1;23(23):15070. doi: 10.3390/ijms232315070 (PMC9735846; doi:10.3390/ijms232315070)
Supplement: Supplementary file 1 [file ijms-23-15070-s001.zip › ijms-1923599-supplementary.pdf]

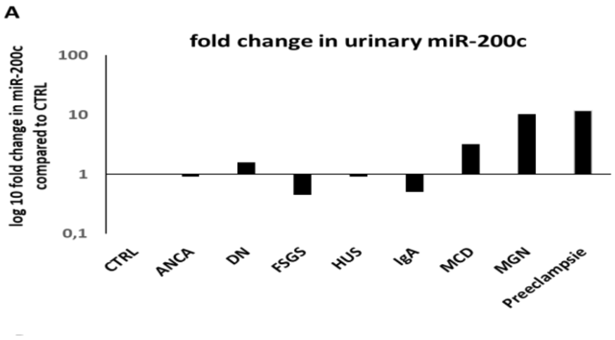

Supplementary Figure S1. Fold change in miR-200c expression in pooled urines from patients with different glomerular diseases. Expression is given as fold change compared to control urine. Urines were normalized to Cel-miR-9, which was spiked into the urines prior to RNA isolation**.**

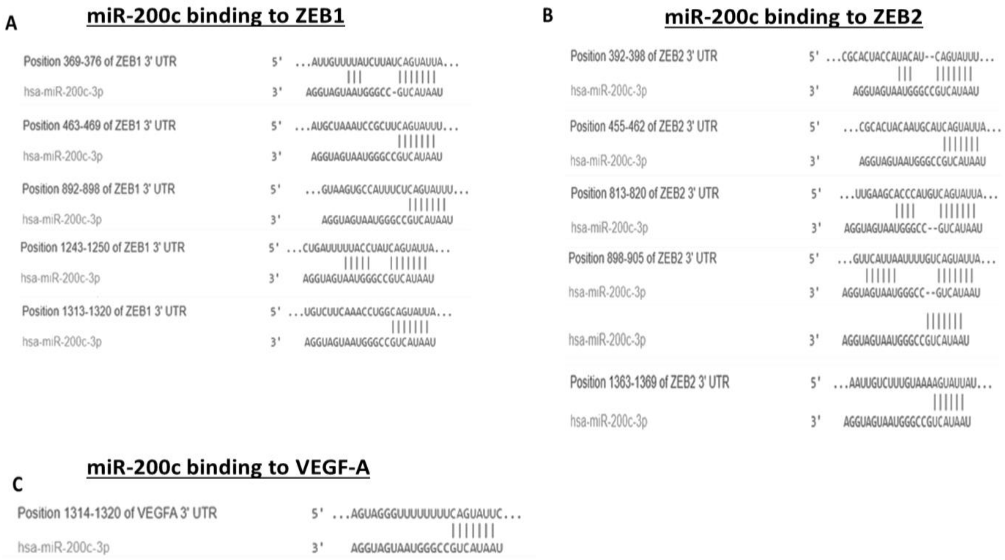

Supplementary Figure S2. Position and binding site of miR-200c to different 3'UTR regions.

Position and binding site of miR-200c to ZEB1 (A), ZEB2 (B) and VEGF-A (C).
